# No evidence of prenatal diversifying selection at locus or supertype levels in the dog MHC class II loci

**DOI:** 10.1186/s40575-016-0038-9

**Published:** 2016-11-18

**Authors:** Alina K. Niskanen, Lorna J. Kennedy, Hannes Lohi, Jouni Aspi, Tanja Pyhäjärvi

**Affiliations:** 1 Department of Genetics and Physiology, University of Oulu, PO Box 3000, Oulu, FIN-90014 Finland; 2 Present address: Centre for Biodiversity Dynamics, Department of Biology, Norwegian University of Science and Technology, NO -7491, Trondheim, Norway; 3 Centre for Integrated Genomic Medical Research, University of Manchester, Stopford Building, Oxford Road, Manchester, M13 9PT UK; 4 Department of Veterinary Biosciences, Research Programs Unit, Molecular Neurology, University of Helsinki and Folkhälsan Institute of Genetics, Biomedicum Helsinki, PO Box 63, FIN-00014 Helsinki, Finland

**Keywords:** *Canis familiaris*, MHC, Prenatal selection, Supertype classification

## Abstract

**Background:**

Despite decades of studying, the mechanisms maintaining high diversity in the genes of the Major Histocompatibility Complex (MHC) are still puzzling scientists. In addition to pathogen recognition and other functions, MHC molecules may act prenatally in mate choice and in maternal-foetal interactions. These interactions are potential selective mechanisms that increase genetic diversity in the MHC. During pregnancy, immune response has a dual role: the foetus represents foreign tissue compared to mother, but histo-incompatibility is required for successful pregnancy. We have studied the prenatal selection in MHC class II loci (DLA-DQA1, DLA-DQB1 and DLA-DRB1) in domestic dogs by comparing the observed and expected offspring genotype proportions in 110 dog families. Several potential selection targets were addressed, including the peptide-binding site, the MHC locus, three-locus haplotype and supertype levels. For the supertype analysis, the first canine supertype classification was created based on *in silico* analysis of peptide-binding amino-acid polymorphism.

**Results:**

In most loci and levels, no deviation from the expected genotype frequencies was observed. However, one peptide-binding site in DLA-DRB1 had an excess of heterozygotes among the offspring. In addition, if the father shared a DLA-DRB1 allele with the mother, that allele was inherited by the offspring more frequently than expected, suggesting the selective advantage of a histo-compatible foetus, in contrast to our expectations.

**Conclusions:**

We conclude that there is some evidence of post-copulatory selection at nucleotide site level in the MHC loci of pet dogs. But due to no indication of selection at locus, three-locus, or supertype levels, we estimated that the prenatal selection coefficient is less than 0.3 in domestic dogs and very likely other factors are more important in maintaining the genetic diversity in MHC loci.

**Electronic supplementary material:**

The online version of this article (doi:10.1186/s40575-016-0038-9) contains supplementary material, which is available to authorized users.

## Plain English summary

Major Histocompatibility Complex (MHC) is a large genetic region coding for immune defense molecules and it is found in all vertebrates. MHC molecules trigger immune response by binding molecules derived from pathogens and recognise self-tissues from non-self-tissues. The MHC molecules are often very diverse. This diversity is mainly thought to be a consequence of balancing selection caused by pathogen pressure. However, also selection before birth that may take place during mate choice or during pregnancy have been demonstrated in some species. Since domestic dogs are often under intense veterinary care, pathogen pressure might be less strong compared to wild animals. Yet, selective patterns of MHC diversity before birth have not been studied before in dogs.

We studied the selection before birth at three MHC class II genes in 10 dog breeds and four mixed-breed groups. We compared e.g. the expected and observed ratios of heterozygous and homozygous puppies in 110 dog families. We conducted selection analyses in many different levels, including: I) sites that bind molecules derived from pathogens (= peptide-binding site), II) the MHC genes, III) three-gene combinations and IV) supertypes. We created the first canine supertype classification that joins different MHC alleles into larger supertypes based on their chemical similarities.

We found that puppies were more heterozygous than expected at one peptide-binding site of one MHC gene (DLA-DRB1). We also found that if the father shared a DLA-DRB1 allele with the mother, that allele was inherited by the offspring more frequently than expected. Other levels of investigation did not show deviations from expected ratios of homozygous and heterozygous offspring. As a conclusion, we found some evidence of selection before birth in dogs. However, other factors are likely contributing more to the MHC diversity in dogs.

## Background

Natural selection in the genes of the Major Histocompatibility Complex (MHC) has been studied extensively in wild animal populations [1]. Across many species, the MHC genes are the most polymorphic in the genome. The main function of classical MHC molecules is to bind antigenic peptides and present them to T-cells [2]. The MHC genes are divided into two classes; class I molecules are expressed on the surface of almost all nucleated cells, while, in man, class II molecules are expressed on specific antigen-presenting cells (e.g. macrophages and lymphocytes). In man, the extended MHC region also includes non-classical genes that have diverse immune and non-immune functions, including the complement system, and olfactory receptor (OR) genes [3, 4].

Since the function of MHC molecules is triggering immune response, parasites and diseases have been thought to play the major role in the maintenance of MHC diversity [5, 6]. However, in domesticated animals pathogen pressure has probably had a smaller contribution to the recent selection targeting MHC, compared to wild species. Instead, there are other potential forces driving selection, such as mate choice, sperm selection, maternal-foetal interactions and autoimmune diseases [7–9]. Many MHC associations with autoimmune diseases have been identified in man [10]. Similar MHC associations have also been demonstrated in canine autoimmune disorders [11]. These associations may be one of the selective forces influencing MHC diversity among dogs (*Canis familiaris*).

The close relationship between the owner and their dog has been a key driver in acquiring extensive knowledge of canine diseases and resulted in a high level of veterinary care, especially in Western societies. The formation of dog breeds and artificial selection by humans has been especially strong during the last 100–150 years. As a consequence, dog breeds have become small and genetically distinct groups. MHC diversity differs from breed to breed and is often low [12], probably due to drift effects in small populations. However, even without free mate choice, MHC diversity in a dog breed can be close to the diversity of a wild wolf population [13, 14]. Humans have had major impact in some of the aspects that may influence the dog MHC diversity, for instance, through veterinary care and mate choice. Therefore, if MHC incompatibility causes prenatal selection, it may be possible to detect such selection in the domestic dog, where other factors of natural selection may be less strong.

### Prenatal selection

Although the overall MHC diversity of dogs has been studied widely [12, 15], the role of prenatal selection in the maintenance of the MHC diversity has not previously been examined. Mate choice in dogs is more often decided by the owner rather than the dog, especially in the purebred dog population. Hence, natural selection may have its strongest impact on the MHC diversity at the post-copulatory stages from egg fertilisation until birth. Association between MHC variation and fertilisation success has been demonstrated in other species, e.g. mice [16]. Prenatal selection can be detected from non-Mendelian proportions of MHC genotypes in the offspring. For instance, the excess of heterozygous offspring [17] and increased foetal loss among MHC-matching couples [8] has been found in humans and in pigtailed macaques [18].

From the mother’s perspective, a foetus corresponds to semi allograft with half of its genome being foreign. To maintain gestation, the immune response of the mother is down-regulated. However, a successful pregnancy requires some degree of maternal immune stimulation, which could be induced by histo-incompatibility (immunological dissimilarity) between the mother and the foetus [19, 20].

MHC molecules have crucial role in successful pregnancy. In humans, most classical MHC molecules are downregulated and only one classical MHC class I gene (HLA-C) and two nonclassical MHC class I genes (HLA-E and -G) are expressed in the foetal side of the placenta, in the extravillous trophoblast (EVT) cells [21–23]. The EVT cells migrate into the mother’s uterus and transform small arteries into large blood vessels to ensure sufficient nourishment of the foetus [24]. The process of transforming the arteries is controlled by the maternal natural killer (NK) cells. Most likely all three MHC molecules, HLA-C, −E and -G, interact with maternal NK cells in regulating placental vascularisation [25]. HLA-C molecule is the only polymorphic MHC molecule expressed in the EVT cells, so the success of pregnancy is influenced by the compatibility of the foetal HLA-C molecules and maternal NK cell receptors. Incongruity of the foetal MHC molecules and maternal NK cell receptors might lead to insufficient blood supply to the placenta which can cause pre-eclampsia, miscarriage and other pregnancy disorders [26].

The expression of MHC genes during pregnancy and mechanisms of maternal-foetal interactions in dogs are largely unknown. It has been shown that some classical class I (DLA-88) and II (DLA-DRA) MHC molecules are not expressed in early canine embryos [27] but there is an increase of MHC class II expressing cells in the uterus of pregnant females [28].

### MHC supertypes

The peptide recognition repertoires of MHC molecules produced by different alleles at the same MHC locus overlap with each other to some extent. As part of the effort to develop epitope-based vaccines with the ability to immunise human populations with diverse MHC genotypes [29], MHC alleles have been grouped into supertypes, based on their structural features or peptide-binding specificity. If the actual differences in pathogen recognition and peptide binding are at the MHC supertype level, they might represent the unit of natural selection and should be taken into account when studying selection in the MHC genes. Until now, supertype classifications have mainly been produced for primates and birds [30–32], but none have been attempted for canines.

### Aims of the study

The aim of this study was to investigate possible prenatal selection in three canine MHC class II loci, DLA-DRB1, DLA-DQA1, and DLA-DQB1, by examining the MHC genotype proportions in dog families. We also analysed the MHC allele composition and heterozygosity of the offspring relative to their mothers, to study potential maternal-foetal interactions. Since successful pregnancy requires histo-incompatibility between the mother and the foetus, we would expect to find an excess of heterozygous offspring under the influence of prenatal selection. Similarly, we would expect that, when parents share the same allele, the offspring will inherit the paternal non-shared allele more often than shared allele from their father. Finally, we compared the functional similarities of the identified canine alleles, and generated the first supertype classification for DLA-DRB1 and DLA-DQB1 alleles.

## Methods

### Samples and DNA techniques

The data comprise 430 puppies and 220 parents in 110 families that belong to ten purebred and four different mixed-breed groups (Table 1 and Additional file 1: Table S1). Each family includes parents and 1–16 puppies with the mean of 3.9 puppies. Apart from a few litters that were fully sampled, a random sample of the offspring was available for most families. Multiple litters from the same parents were combined as one litter. The dogs were sampled in Finland, UK and USA. Thirty-six individuals were in the dataset both as a parent and as an offspring. Thirty parents had more than one litter with different mates (24 parents had two, four had three, and two had four litters).

**Table 1:** The dog breeds, the number of offspring (N_O_), the number of families (N_F_), the country of sampling and the MHC genotyping method

Breed	N_O_	N_F_	Country^a^	MHC genotyping method
Kooikerhondje^b^	17	10	Finland	Sequencing
Saluki^b^	51	16	Finland	Sequencing
Lowchen^b^	9	4	Finland	Sequencing
Icelandic Sheepdog^b^	7	3	Finland	Sequencing
Kromfohrlander^b^	90	24	Finland	Sequencing
Alaskan Husky^c^	27	6	USA	Sequencing
Alaskan Husky^d^	4	1	USA	Sequencing
English Cocker Spaniel^d^	6	1	USA	Sequencing
Golden Retriever^d^	7	3	USA	Sequencing
Staffordshire Bull Terrier^d^	7	3	USA	Sequencing
Newfoundland^d^	42	12	UK	Sequencing
Rhodesian Ridgeback^d^	42	5	UK	Sequencing
Mixed-breed^d^	84	16	USA	Sequencing
Mixed-breed^c^	37	6	USA	Sequencing or microsatellite based
Total	430	110		

^a^Birth country may differ from the country of sampling, ^b^Data from this study, ^c^Rowan [35], ^d^Kennedy et al. [34]

The highly polymorphic second exon that encodes the peptide binding part of the MHC molecule was sequenced for DLA-DRB1 (270 bp), DLA-DQA1 (246 bp) and DLA-DQB1 (267 bp) genes. The genes are located close together on canine chromosome 12 in the above-mentioned order [33]. The distance between DLA-DRB1 and DLA-DQA1 is 56.6 kb, while the distance between DLA-DQA1 and DLA-DQB1 is 77.7 kb. A previously established protocol [13] was used to sequence the loci in a cohort of 253 dogs. In addition, data from 41 families with 192 puppies from an earlier study by Kennedy et al. [34] plus a further 12 families with 64 puppies from Rowan [35] were included. Microsatellite genotyping method for MHC genotyping was used for one mixed-breed group of six families including 37 offspring [35]. In this method, dogs were genotyped using microsatellites CFA12-3 (dinucleotide), CFA12-4 (tetranucleotide), and CFA12-9 (dinucleotide), which are linked with the studied DLA loci [36]. In addition, parental dogs were genotyped by sequencing to confirm the MHC genotypes.

### MHC allele and haplotype assignment

MHC alleles were determined from the sequence data using MatchTools and MatchTools Navigator (Applied Biosystems) programs. MatchTools includes a library of canine MHC alleles and compares a homozygous or heterozygous sequence pair to the allele sequences in the library. Two puppies from the data by Kennedy et al. [34] had a recombinant allele in one locus and that locus was excluded from the analyses in those individuals.

Usually, the MHC alleles from different loci are inherited as haplotypes due to strong linkage disequilibrium (LD, non-random association of alleles between different loci). Three-locus haplotypes consisting of the DLA-DRB1, DLA-DQA1 and DLA-DQB1 alleles were defined in three steps: 1) haplotypes were determined for homozygous individuals, 2) haplotypes for heterozygous individuals were inferred based on the haplotypes observed in the homozygous individuals and 3) rare haplotypes, detected only in the heterozygous individuals, were identified by subtraction of haplotypes determined in the previous steps [14]. In 35 families, missing parental haplotypes were inferred based on the haplotypes of the offspring and the other parent. The DLA-DQA1 alleles of three parents and the DLA-DQB1 allele of one parent were inferred based on the family members’ alleles.

In the microsatellite-based genotyping of the DLA alleles, the microsatellite data were analysed with Genotyper 3.7 (Applied Biosystems). The microsatellite alleles were matched with the DLA haplotypes in the dogs that had been genotyped by both methods. After identifying the corresponding microsatellite alleles and DLA haplotypes, the DLA haplotypes were assigned to individuals that had been genotyped only with microsatellites.

### MHC supertype classification

MHC supertype classification was developed to find out if prenatal selection acts at that level. MHC alleles in the β loci, DLA-DRB1 and DLA-DQB1, were classified into MHC supertypes based on the chemical properties of the amino acid sequences in the putative peptide-binding region (PBR). The classification was done for officially named canine MHC alleles from the MatchTools library comprising alleles from different canine species, mostly from dog and grey wolf (*Canis lupus*). Altogether 205 DLA-DRB1 alleles and 100 DLA-DQB1 alleles were included.

Since the supertype classification of alleles is based on the shared properties in peptide binding, the putative PBR sites were selected according to human PBR sites proposed by Doytchinova and Flower [37]. Only polymorphic PBR sites were included in the analyses; 19 sites in DLA-DRB1 (codon positions: 9, 11, 13, 26, 28, 30, 38, 47, 56, 57, 60, 61, 67, 70, 71, 74, 78, 86 and 90) and 15 sites in DLA-DQB1 (codon positions: 9, 13, 26, 28, 30, 37, 38, 47, 57, 67, 70, 71, 74, 77 and 85). Also 37 canine DLA-DQA1 alleles were checked for polymorphisms in putative PBR sites but all 10 sites were monomorphic and thus the locus was excluded from the supertype classification.

Chemical properties of the amino acids in PBR sites were described using five z-descriptors: z_1_ (hydrophobicity), z_2_ (steric bulk), z_3_ (polarity), z_4_ and z_5_ (electronic effects) [38]. Z-descriptor values were standardised so that their distributions had a mean of zero and a standard deviation of one. All z-descriptor × polymorphic site combinations were used for estimating Euclidian distances between the different MHC alleles. Based on the Euclidian distances, the alleles were clustered using hierarchical clustering with Ward’s method [39] in R statistical environment v. 3.1.2 [40]. The number of clusters was decided based on the cost of merging clusters in Ward’s method. The smallest number of clusters, before a steep increase in the cost of merging clusters (variance within clusters), was chosen. To study which PBR sites had most influence in the clustering and to visualise the relations of the supertype clusters, a principal component analysis (PCA) was conducted using the *prcomp* function in R.

### Segregation analyses

To study whether the DLA-DRB1, DLA-DQA1 and DLA-DQB1 alleles follow Mendelian inheritance in dog families, heterozygosity analyses were conducted in R statistical environment v. 3.1.2 [40]. There are four possible mating cross types where differences in expected and observed numbers of heterozygotes can be observed: A_1_A_1_ × A_1_A_2_, A_1_A_2_ × A_1_A_1_, A_1_A_2_ × A_1_A_2_ and A_1_A_2_ × A_1_A_3_ (where: mother × father, and the alleles A_1_ ≠ A_2_ ≠ A_3_ are composites of different observed alleles). In our dataset, altogether 74 (DLA-DRB1 and DLA-DQB1) and 83 (DLA-DQA1) families including 293 (DLA-DRB1), 313 (DLA-DQA1) and 295 (DLA-DQB1) puppies met this criterion. The numbers of heterozygous and homozygous offspring were counted within each mating cross type. The expected numbers of heterozygous and homozygous offspring were calculated based on the Mendelian inheritance model and compared to observed numbers of offspring using Pearson’s chi-squared test with Monte Carlo simulated *p*-value (2 000 repeats). Chi-squared test statistics were also combined over the mating cross types in each locus to examine the overall deviation from the expected number of the heterozygotes.

The analyses were conducted at locus and three-locus haplotype levels for the entire data, and separately for different breeds/groups and sexes to identify the potential breed or sex related differences in the inheritance patterns. Information about sex was available for 381 offspring. In addition, purebred and mixed-breed dogs were analysed separately. Segregation analyses were also done for the supertype classes and separately for each polymorphic PBR site in the DLA-DRB1 and DLA-DQB1 loci. To correct for multiple testing, a false discovery rate (*q*-value [41]) was estimated for *p*-values of the combined chi-squared tests of the different mating cross types over the PBR sites in one locus.

Two haplotypes that were found in four breeds/groups carried a duplicated DLA-DQB1 locus (DLA-DQB1*01303 and DLA-DQB1*01701 [34]). Since it is unclear whether both copies are expressed or not, analyses were conducted including all individuals and separately for the families with (number of puppies = 51 (DRB1)/59 (DQA1)/53 (DQB1)) and without (number of puppies = 242 (DRB1 & DQB1)/254 (DQA1)) the duplicated locus. When included in the analyses, the duplicated locus was handled as one allele (DLA-DQB1*013017). In the analyses for the DLA-DQB1 locus with supertype classification and for each PBR site, all families with duplicated locus were excluded.

### Maternal effect

We studied the maternal effect on the MHC class II allele transmission in the families where the offspring may inherit the maternal shared or paternal non-shared allele from their father: A_1_A_2_ × A_1_A_3_ and A_1_A_1_ × A_1_A_2_. We tested the distribution between the expected and observed numbers of the shared and non-shared alleles inherited from father with Pearson’s chi-squared test with Monte Carlo simulated *p*-value (2 000 repeats).

There is some evidence in man, that the mother’s heterozygosity may influence the offspring heterozygosity – in Amerindian families only the heterozygous mothers’ had increased proportion of heterozygous offspring [17]. So, to discover whether the mother’s heterozygosity has an effect on the heterozygosity of the offspring in dogs, families with heterozygous mothers were joined for analyses. The maternal effect was studied in the total dataset and in the same subsets as described in section 2.4.

## Results

### MHC alleles and haplotypes

Altogether 28 DLA-DRB1, 11 DLA-DQA1 and 23 DLA-DQB1 alleles organised in 45 three-locus haplotypes were found in the dog sample (Table 2). There were some common haplotypes that were found in about half of the breeds/groups studied but rare unique haplotypes were also detected in many breeds. Three new DLA-DRB1 alleles were found among the 253 dogs sequenced for this study. The allele sequences have been submitted to NCBI GenBank with the accession numbers: JQ904819.1, FM246834, and FM246838.

**Table 2:** Three-locus MHC haplotypes and their frequencies in the studied dog breeds and mixed-breed groups

DRB1	DQA1	DQB1	AH^1^	AH^2^	CB^1^	CB^2^	ECS^2^	GR^2^	ISD	KKH	KL	LW	NF^2^	RRB^2^	SA	SBT^2^
10301	00101	00802									0.28					
00101	00101	00201	0.15		0.24	0.22			0.08					0.30	0.02	0.35
00101	00101	03601										0.12			0.02	
00101	00101	03603										0.12				
00103	00101	00201				0.06										
00201	00901	00101		0.25									0.17			
00601	005011	00701	0.17	0.25		0.01	1.00						0.08	0.18	0.05	
00601	005011	02001						0.12					0.33			
00901	00101	008011	0.32							0.42	0.03	0.47			0.02	
010011	00201	01501				0.06										
01101	00201	01303								0.04						
01102	00201	01303							0.08							
01201	00401	013017^a^		0.33	0.51								0.19		0.01	
01201	00401	01303						0.42								
01301	00101	00201							0.19				0.04	0.25		0.46
01301	00601	02002													0.07	
01501	00901	00101														0.19
01501	00601	02301	0.24			0.31		0.19	0.23	0.03			0.04			
01501	00601	05701	0.03													
01501	00601	00301							0.12							
01501	00601	02201									0.30					
01501	00601	02002				0.07										
01501	00401	013017^a^													0.03	
01501	012011	03501													0.01	
01501	00601	01101								0.18		0.09				
01502	00601	02301									0.22	0.03				
01503	00601	00301							0.08							
01503	00601	02304								0.07						
01801	00101	00802	0.03		0.24									0.14	0.07	
02001	00401	01303						0.27		0.09			0.04	0.01	0.42	
02301	00301	00501							0.23	0.18						
03001	00601	00301											0.03			
04001	01001	01901		0.17												
07001	01801	05001	0.06													
07401	005011	00701									0.16				0.02	
07401	00101	00201													0.01	
07601	00601	02301				0.25										
07801	00401	01303													0.08	
08001	00402	01303													0.01	
09401	00101	008011										0.18				
09501	00301	05401													0.05	
09501	00101	008011													0.02	
09501	00901	00101													0.02	
09701	00601	02002													0.04	
09801	00402	02301													0.01	
		NA				0.02							0.08	0.12	0.01	
		*n*	39	6	49	116	8	13	13	37	138	17	66	52	83	13

*AH* ^*1*^ Alaskan Husky, *AH* ^*2*^ Alaskan Husky, *CB* ^*1*^ Mixed-breed, *CB* ^*2*^ Mixed-breed, *ECS* ^*2*^ English Cocker Spaniel, *GR* ^*2*^ Golden Retriever, *ISD* Icelandic Sheepdog, *KKH* Kooikerhondje, *KL* Kromfohrlander, *LW* Lowchen, *NF* ^*2*^ Newfoundland, *RRB* ^*2*^ Rhodesian Ridgeback, *SA* Saluki, *SBT* ^*2*^ Staffordshire Bull Terrier

*n* = number of individuals (all litter-parents combinations)

^a^ Duplicated DLA-DQB1 locus, alleles DLA-DQB1*01303 and DLA-DQB1*01701

^1^ Rowan [35], ^2^ Kennedy et al. [34]

### Supertype classification

**Fig. 1:**
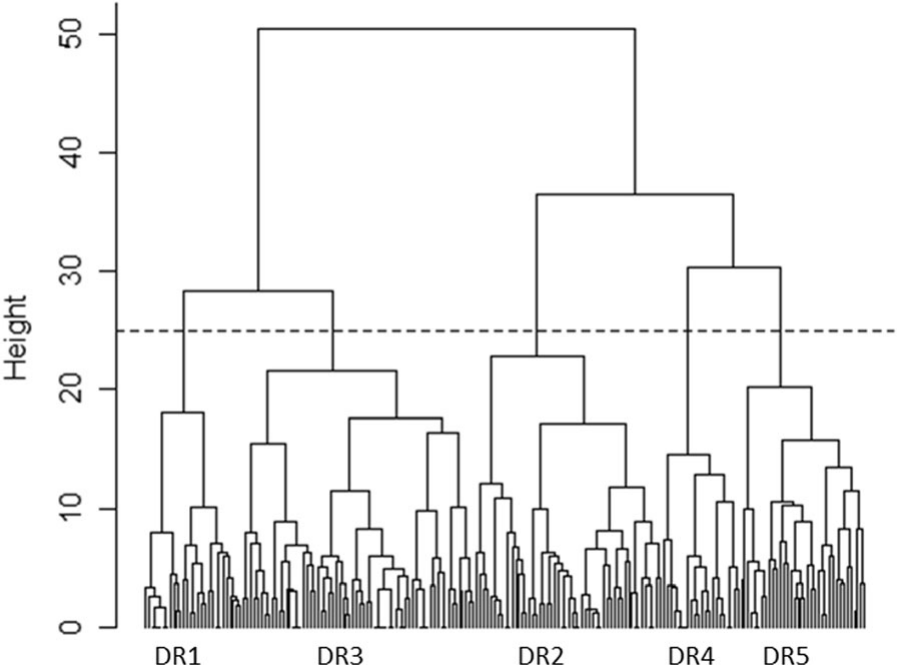
Supertype clustering tree for the canine DLA-DRB1 alleles. Dashed line indicates division into the five supertype classes

**Fig. 2:**
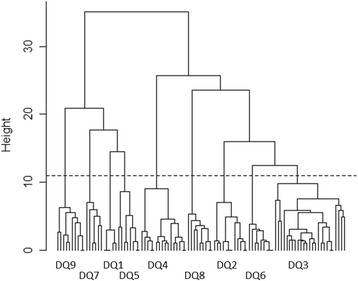
Supertype clustering tree for the canine DLA-DQB1 alleles. Dashed line indicates division into the nine supertype classes

The DLA-DRB1 alleles were divided into five supertype classes and the DLA-DQB1 alleles were divided into nine supertype classes using hierarchical clustering method. The number of classes was based on the cost of merging clusters (Additional file 2: Figures S1–S2). Supertype clustering trees for the canine DLA-DRB1 and DLA-DQB1 alleles are shown in Figs. 1 and 2 and the supertype clustering trees with allele names are shown in Additional file 3: Figures S3–S4. Complete lists of the alleles in each supertype class are in Additional file 4: Table S2.

In humans, each MHC supertype could be described by common fingerprint amino acids that were shared within each supertype class [37]. In canines, such supertype fingerprints could not be detected, since amino acids were shared, to some extent, also between different supertypes. Based on the three first PCs the PBR sites 1, 5, 6, and 9 in DLA-DRB1, and 4, 9, 10, 12, and 15 in DLA-DQB1 had the largest impact on the supertype clustering (PC1 vs. PC2 in Additional file 5: Figures S5–S6). At these sites, there were from two to seven (DLA-DRB1) and from three to five (DLA-DQB1) different amino acids – a similar level of diversity compared to other PBR sites.

### MHC genotype proportions in the offspring

**Fig. 3:**
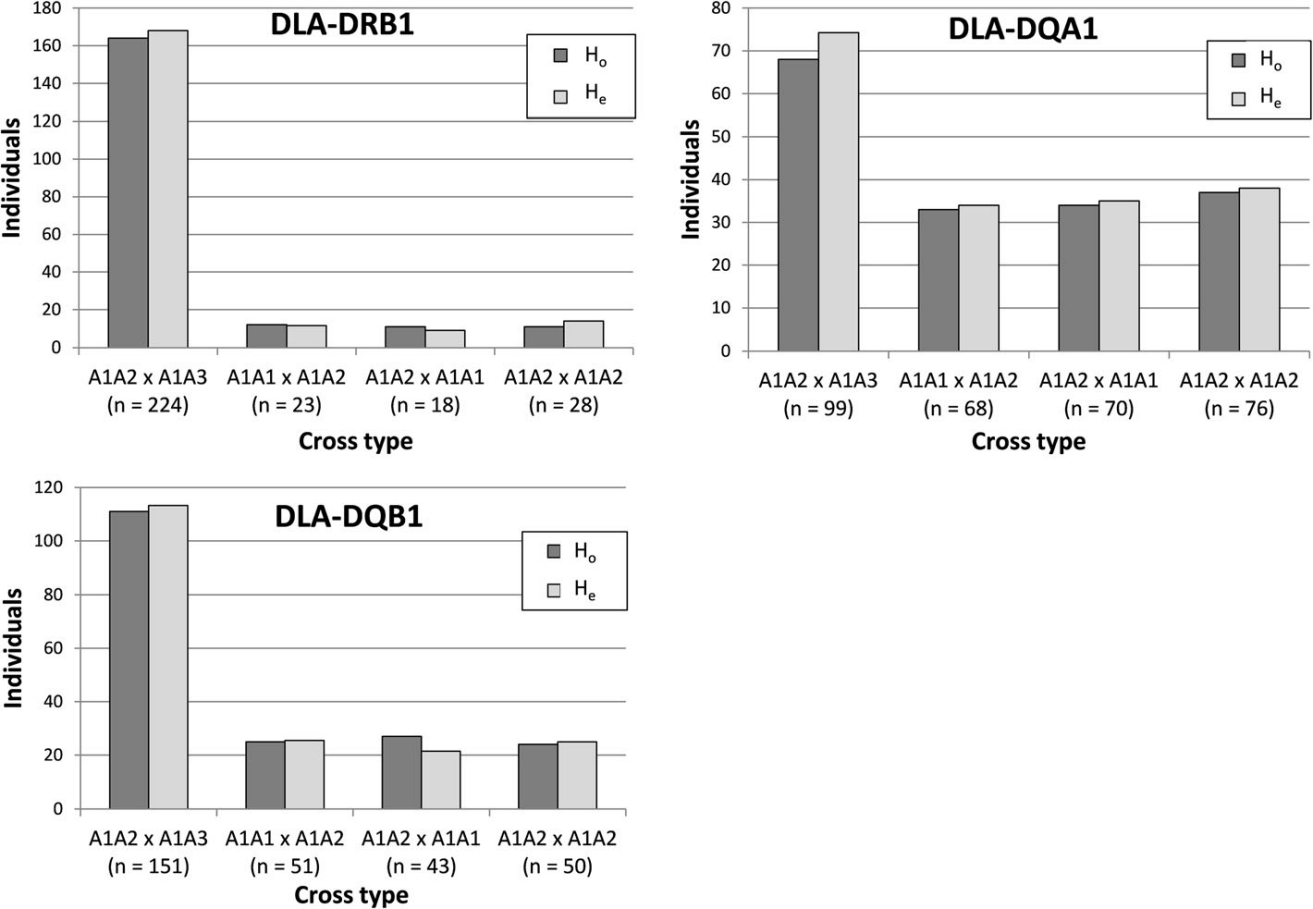
The observed (H_o_) and expected (H_e_) numbers of the heterozygous offspring from different mating cross types for the DLA-DRB1, DLA-DQA1 and DLA-DQB1 loci (*n* = total number of offspring within the cross type)

**Fig. 4:**
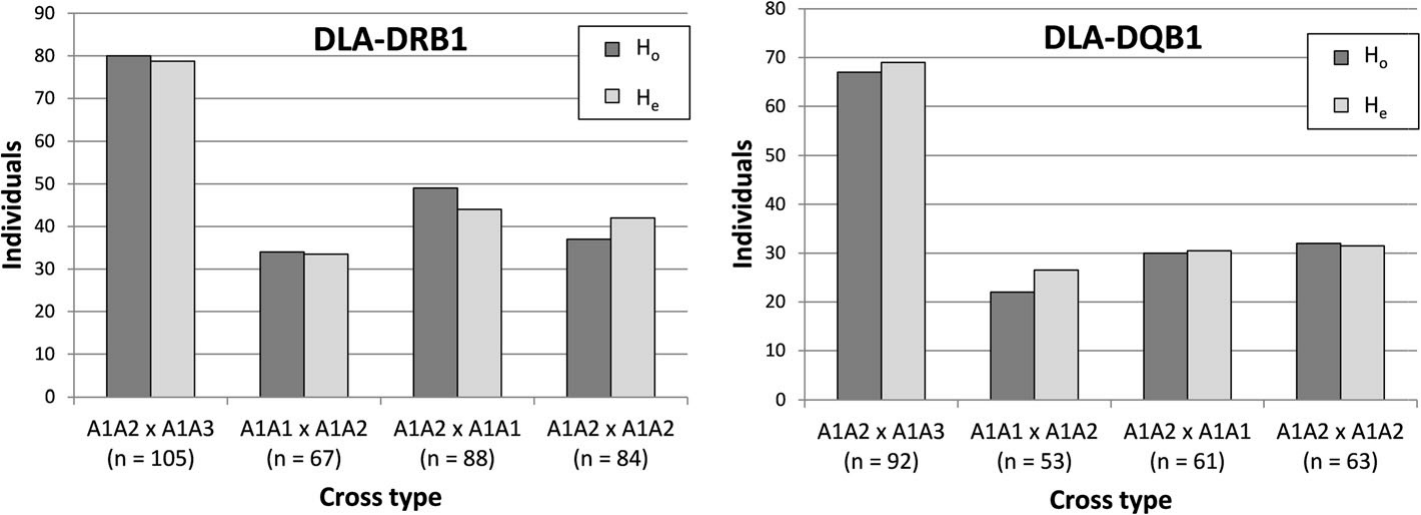
The observed (H_o_) and expected (H_e_) numbers of the heterozygous offspring from different mating cross types for the DLA-DRB1 and DLA-DQB1 supertypes (*n* = total number of offspring within the cross type)

At the locus level, deviation from the Mendelian expectations was not observed in the genotype proportions of the dog families. When examined for all of the families and different mating cross types, *χ* ^*2*^ varied between 0.02 and 2.81 (df = 1) and *p*-value varied between 1 and 0.13 in all MHC loci (Fig. 3 and Additional file 6: Table S3). Accordingly, the combined chi-squared analyses over mating cross types within each locus did not show any deviation from the expected heterozygosity and the combined *χ* ^*2*^ varied between 2.28 and 3.09 (df = 1) and *p*-value varied between 0.69 and 0.54. All of the MHC loci followed the Mendelian inheritance also in different breeds, in purebreds, in mixed-breed dogs, in both sexes and in families with or without the duplicated DLA-DQB1 locus, when they were analysed separately. No deviation from the expected heterozygosity was detected either at the three-locus haplotype level (Additional file 6: Table S3) or using supertype classification (Fig. 4).

**Fig. 5:**
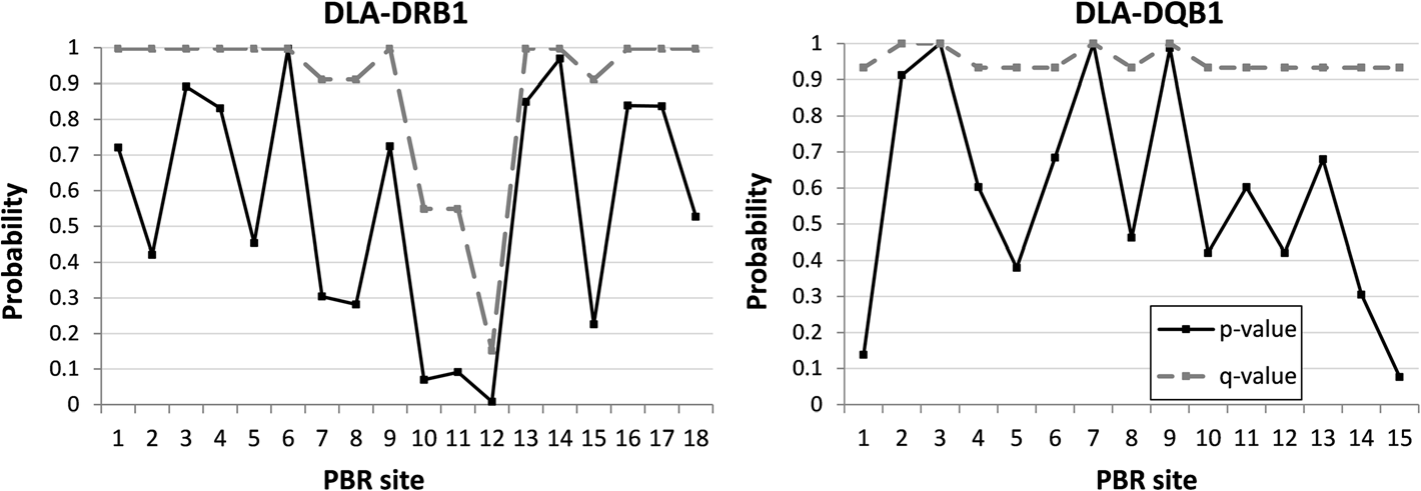
The *p*- and *q*-values for the combined chi-squared statistics testing the ratio of the observed and expected heterozygous offspring for each PBR site in the DLA-DRB1 and DLA-DQB1 loci

The genotype proportions in the offspring were also studied in each polymorphic PBR site. Figure 5 shows both *p*- and *q*-values for the combined chi-squared statistics over the different mating cross types in site-wise segregation analyses. After correcting for multiple testing, the PBR sites 10–12 in DLA-DRB1 showed a slight deviation from the expected heterozygosity, with site 12 having the smallest *q*-value (*q* = 0.15). The PBR sites 10, 11 and 12 correspond to codon positions 57, 60 and 67 in the data. More detailed examination of the PBR site 12 revealed excess of heterozygous offspring from the mating cross type A_1_A_2_ × A_1_A_1_ (*χ* ^*2*^ = 9.48, df = 1, *p* = 0.004). The site was polymorphic for three amino acids: leucine, isoleucine and phenylalanine. All three amino acids are hydrophobic, but phenylalanine differs from the other two by having an aromatic side chain. Also the PBR site 10 showed excess of heterozygosity (A_1_A_2_ × A_1_A_1_, *χ* ^*2*^ = 4.46, df = 1, *p* = 0.04) but the PBR site 11 showed excess of homozygosity (A_1_A_2_ × A_1_A_2_, *χ* ^*2*^ = 4.95, df = 1, *p* = 0.04). In DLA–DQB1, no PBR sites showed deviation from the expected heterozygosity (Fig. 5).

### Maternal effect

**Fig. 6:**
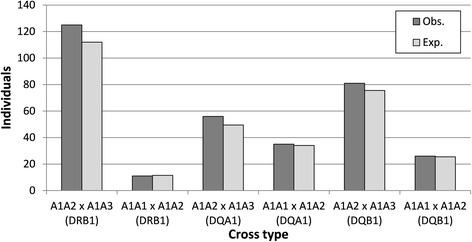
The observed (Obs.) and expected (Exp.) numbers of offspring that inherited the maternal shared allele from their father for DLA-DRB1, DLA-DQA1 and DLA-DQB1 loci

To study the putative prenatal maternal effect, allele transmission was studied in the families in which parents shared one allele and the offspring could inherit the maternal shared or paternal non-shared allele from their father. The puppies tended to inherit the maternal shared allele (A_1_) from their father for DLA-DRB1 in mating cross type A_1_A_2_ × A_1_A_3_ more often than expected (*χ* ^*2*^ = 3.02, df = 1, *p* = 0.09; Fig. 6 and Additional file 6: Table S3). This type of mating included the majority of the families in this analysis. However, no strong deviations from the expected allele proportions were found either when using the allele or supertype classification in the analyses. The maternal shared and non-shared allele were equally often inherited from the father also in different breeds, in both sexes and in families with or without the duplicated DLA-DQB1 locus.

When the heterozygous mothers were combined for segregation analyses, mother’s heterozygosity did not affect the offspring heterozygosity neither at the allele nor at the supertype level (Additional file 6: Table S3).

## Discussion

We have studied prenatal selection in dogs by analysing the segregation of the MHC class II alleles in purebred and mixed-breed families. Natural selection targeting the MHC may be limited in the pet dogs due to their protected environment. One potential phase of natural selection is during foetal development which is less strongly affected by human action or breeding choices. However, we did not detect deviations from the Mendelian segregation pattern at allele, three-locus haplotype, or supertype levels.

At least around 200 DLA-DRB1, 100 DLA-DQB1 and 40 DLA-DQA1 alleles have been found in canines. Obviously there is some functional overlap in such a large number of alleles. In humans, it has been shown that the MHC class II alleles have more shared peptide binding repertoire than the MHC class I alleles [42]. Clustering the MHC alleles into supertypes is one way to reach a meaningful level of functionality and variation for studying the natural selection in the MHC. We have generated the first canine supertype classification for the DLA-DRB1 and DLA-DQB1 alleles. The DLA-DQA1 alleles were not included, since they were not polymorphic on the putative PBR sites proposed by Doytchinova and Flower [37]. This might have only minor effect to our results, since in humans it has been shown that the HLA-DQA1 alleles did not contribute to the supertype clustering when they were analysed together with the HLA-DQB1 alleles to compose a single functional unit [37]. In this study, the number of the supertype clusters (five and nine) was relatively low, and based on *in silico* analysis of amino-acids. Functional studies, where the peptide binding repertoires of the different MHC alleles could be tested, would greatly benefit the extraction of a biologically meaningful number of supertype clusters [31, 42].

The supertype-clustered MHC alleles can be used in selection analyses instead of the sequence-based alleles. However, we did not find patterns suggesting prenatal selection at supertype level in the studied dog families. Previously, natural selection has been detected at the supertype level e.g. in the higher male heterozygosity of newborn humans [43] and in the non-random association of the alleles within DRB-haplotypes of baboons [31]. Parasite and disease resistance has also been associated with MHC supertype level classes in various species [30, 32]. These findings suggest that supertype classification reaches a level of functionality that may be missed using individual alleles in the selection analyses.

In humans, some small isolated populations have shown non-Mendelian proportions of MHC genotypes in offspring [17] or MHC associated foetal loss [8]. However, often in studies of larger human populations no deviation from the expected proportions has been found in the MHC loci [1, 44]. The lack of selection in the modern large human populations might be a consequence of improved health care [45] which also applies to dogs but is likely to mostly affect selective factors after birth and to less affect maternal-foetal interactions. In this study, the puppies were sampled in varying ages and the age of the dog was not known in all cases. Thus, it is possible that a small degree of postnatal selection has occurred.

We found that the dog puppies were more heterozygous than expected in respect to the PBR-site 12 of DLA-DRB1 for the mating cross type A_1_A_2_ × A_1_A_1_. When half of the offspring are expected to be heterozygous and half homozygous, the selection coefficient (*s*) can be estimated with:

$$ s = 1 - {N}_{ii}\ /\ \left({N}_{ij} + 1\right) $$

where *N*_*ii*_ is the observed number of the homozygous offspring and *N*_*ij*_ is the observed number of the heterozygous offspring [46]. The observed excess of the heterozygous individuals at the PBR-site 12 of DLA-DRB1 would require strong selection against homozygotes: *s* = 1 – 38/(70 + 1) = 0.465. Selection of similar strength, *s* = 0.462 over two MHC loci, has been estimated to favor heterozygotes in human populations [17]. However, because the MHC expression in canine fertilisation and pregnancy is not well known, further studies are required to investigate the exact cause of the observed deviation, before reaching further conclusions about the selective advantage of heterozygosity on the PBR-site 12. The amino acids at the PBR-site 12 were shared by different alleles and supertypes, which explains why we did not detect the signal of this deviation at the higher levels of analyses.

Our data consisted of ten dog breeds, that have been bred for e.g. hunting, herding, and companion purposes, and mixed-breed groups. The sampling of numerous breeds had the advantage of representing the species more widely, but as a downside, it also caused individual alleles to have low frequencies in the highly polymorphic MHC loci. To gain statistical power, we classified the mating cross types by the alleles the parents shared without keeping the information of allele identity. Simultaneously, we compromised the putative effects of individual alleles. We estimated the statistical power of the *χ* ^*2*^ test to distinguish between the expected and observed numbers of puppies at the *p* = 0.05 level. Using the sample sizes shown in Fig. 3 for each mating cross type and locus, *s* should approximately be above 0.3–0.6 to be detected. Values this high indicate very strong selection and are rarely observed in natural populations. It is possible that there are breed-specific differences, which we were not able to recognise due to the small breed-wise sample size, particularly when the sample was further divided into mating cross types. As shown in earlier studies [17], small sample size can hinder us from drawing final conclusions about the differences in the post-copulatory selection in dog breeds, especially if selection is not very strong.

Due to the high LD within the MHC region, MHC class II alleles could show the effect of prenatal selection even if the class II alleles are not expressed in the placenta. Other prenatally selected loci that are in LD with the classical MHC loci may contribute to the maintenance of the high polymorphism in the extended MHC region [47]. Simultaneously, this may lead to apparent segregation distortion also in the classical MHC genes. For example, Laurent et al. [48] detected a signature of disassortative mating using single nucleotide polymorphisms (SNPs) in the extended human MHC region (3.6 Mb), and concluded that it was caused by multiple MHC related genes rather than the classical MHC genes. Mate choice is seldom possible in dogs, but post-copulatory selection may, in a similar way, be caused by several MHC-linked genes.

The OR genes may influence both mate selection and the success of spermatozoa in the fertilisation [47]. Previously observed non-Mendelian segregation of MHC alleles in primates may be caused by selection targeted to MHC-linked OR genes. If that was the case, the missing selection in the MHC class II loci of our dog families is easily explained by the fact that no OR genes have this far been localised in the major 3 Mb MHC region on the dog chromosome 12 or elsewhere on chromosome 12 [33, 49]. Dog spermatozoa do express OR genes [50], but putatively MHC-linked OR genes are only found in the minor 600 kb MHC class I region located on dog chromosome 35 [33].

## Conclusions

In summary, we found some evidence of post-copulatory selection in pet dogs at nucleotide site level, but not at locus, three-locus, or supertype levels in MHC loci. It is possible that there is weaker selection affecting other sites and levels, but our sample, which only spanned one generation, was not large enough to pick up the signal [1]. Since selection has modified the MHC diversity over a long time period, even weak selection may have been sufficient to preserve the diversity.

To our knowledge, this was the first study of prenatal selection in dog MHC loci and we have some suggestions how to proceed with future studies. In further examinations of MHC related prenatal selection in canines, a comprehensive study including mate choice, segregation of alleles and litter size would be interesting. Future studies would also benefit from using dogs from more primitive circumstances and, for comparison, their wild wolf relatives.

## Additional files

Additional file 1: Table S1. MHC genotype data and sample information for the dog families. (XLSX 53 kb)
Additional file 2: Figures S1 and S2. Merging cost showing the amount of variance within clusters in Ward’s method. This information was used in deciding the number of supertype clusters in DLA-DRB1 (**Figure S1**) and DLA-DQB1 (**Figure S2**) loci. (ZIP 3 kb)
Additional file 3: Figures S3 and S4. Supertype clustering trees for the canine DLA-DRB1 (**Figure S3**) and DLA-DQB1 (**Figure S4**) loci with allele names included. The supertype clusters are coloured differently. (ZIP 12 kb)
Additional file 4: Table S2. Supertype classification allele data and all alleles in the dog family data. (XLSX 25 kb)
Additional file 5: Figures S5 and S6. Biplots of the first two PCs in supertype clusters of DLA-DRB1 (**Figure S5**) and DLA-DQB1 loci (**Figure S6**). The biplots were done using the *ggbiplot* function in R. Ellipses show Normal contour lines with 68% probability for each cluster. (ZIP 20 kb)
Additional file 6: Table S3. Result table for segregation of heterozygous and homozygous offspring in different mating cross types for the MHC loci, the three-locus haplotype and the MHC supertype levels, heterozygous mothers combined and the numbers of offspring that inherited maternal shared or paternal non-shared allele from their father. (XLSX 12 kb)

## Abbreviations

df: degree of freedom
DLA: Dog leucocyte antigen
DNA: Deoxyribonucleic acid
EVT: Extravillous trophoblast cells
*F*_*IS*_: Inbreeding coefficient
*H*_*e*_: Expected number of heterozygotes
HLA: Human leucocyte antigen
*H*_*o*_: Observed number of heterozygotes
LD: Linkage disequilibrium
MHC: Major histocompatibility complex
*N*_*F*_: Number of families
NK: Natural killer cells
*N*_*O*_: Number of offspring
*OR*: Olfactory receptor
PBR: Peptide-binding region
PC(A): Principal component (analysis)
*s*: selection coefficient
