# Supplementary figures and images for: No evidence of prenatal diversifying selection at locus or supertype levels in the dog MHC class II loci
Source: Canine Genet Epidemiol. 2016 Nov 18;3:9. doi: 10.1186/s40575-016-0038-9 (PMC5116190; doi:10.1186/s40575-016-0038-9)

# Cost of merging DLA-DRB1 supertype clusters Ward's method

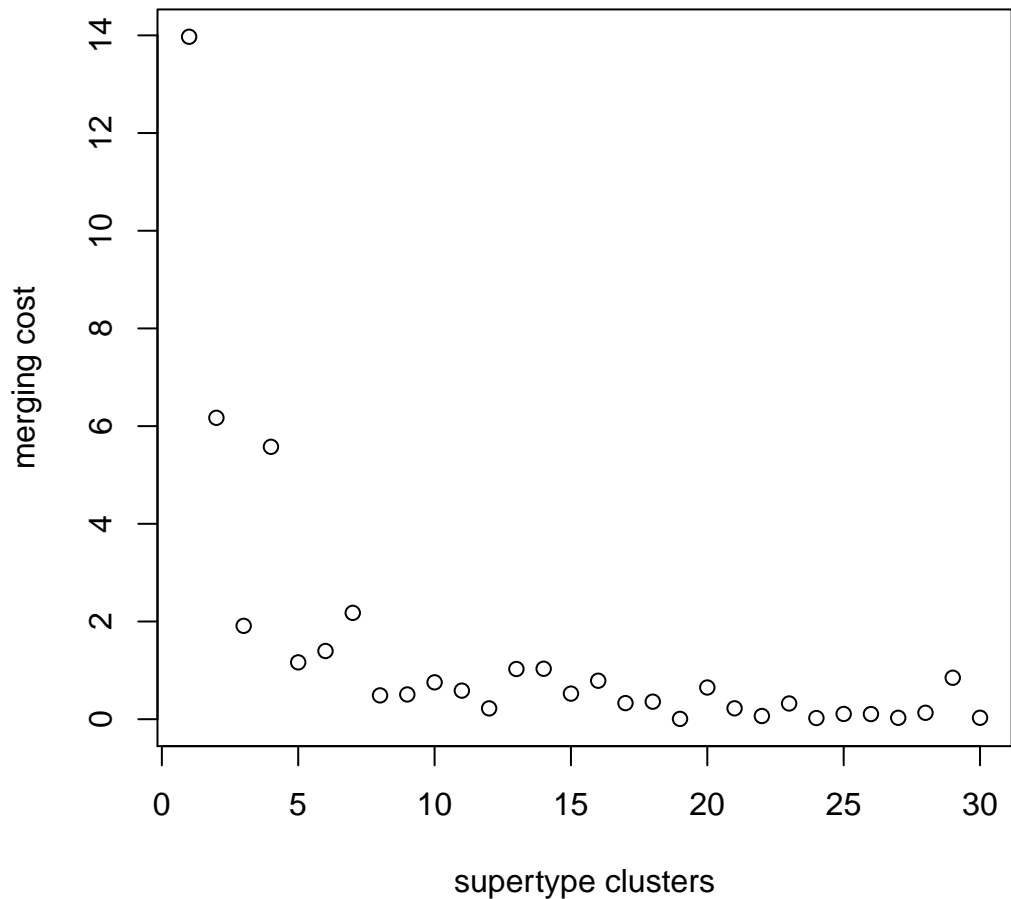

Supplement: Additional file 2: Figures S1 and S2. — Merging cost showing the amount of variance within clusters in Ward’s method. This information was used in deciding the number of supertype clusters in DLA-DRB1 (Figure S1) and DLA-DQB1 (Figure S2) loci. (ZIP 3 kb) [file 40575_2016_38_MOESM2_ESM.zip › Supplement figS1 DRBMergingCost30Clusters.pdf]

# Cost of merging DLA-DQB1 supertype clusters Ward's method

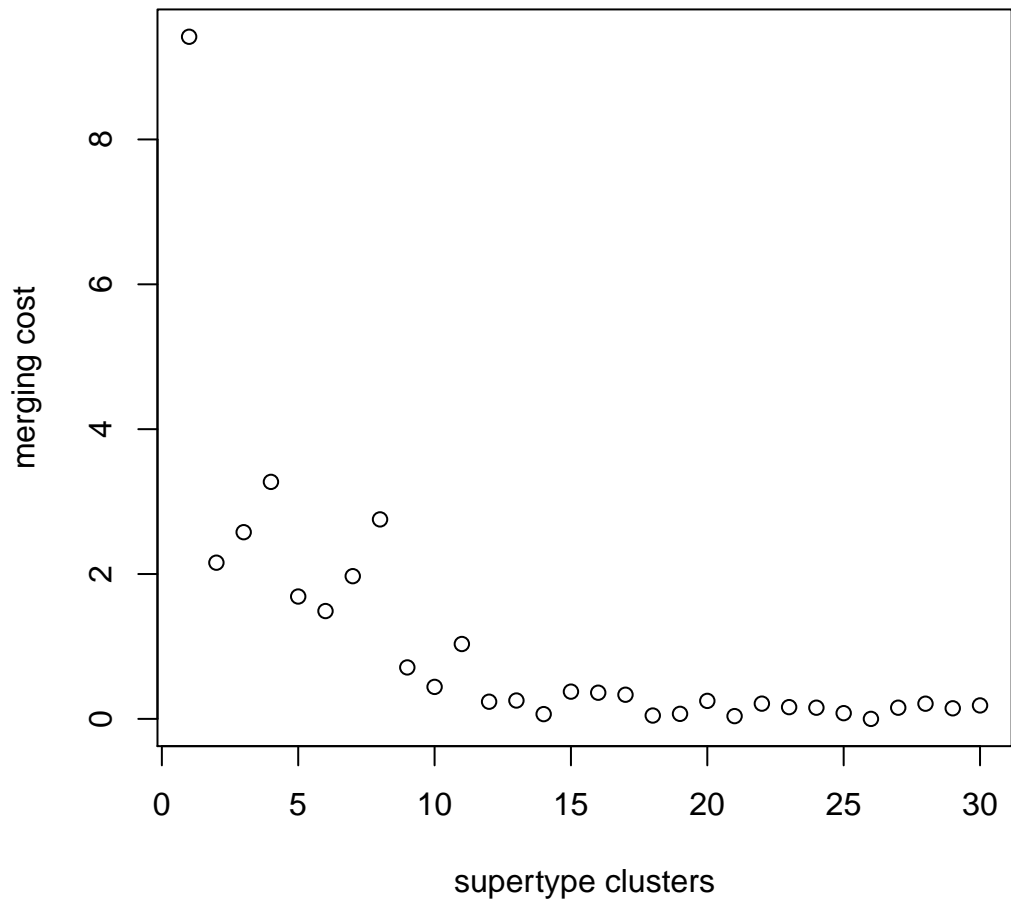

Supplement: Additional file 2: Figures S1 and S2. — Merging cost showing the amount of variance within clusters in Ward’s method. This information was used in deciding the number of supertype clusters in DLA-DRB1 (Figure S1) and DLA-DQB1 (Figure S2) loci. (ZIP 3 kb) [file 40575_2016_38_MOESM2_ESM.zip › Supplement figS2 DQB merging cost in Ward.pdf]

DLA-DRB1 supertype clustering tree

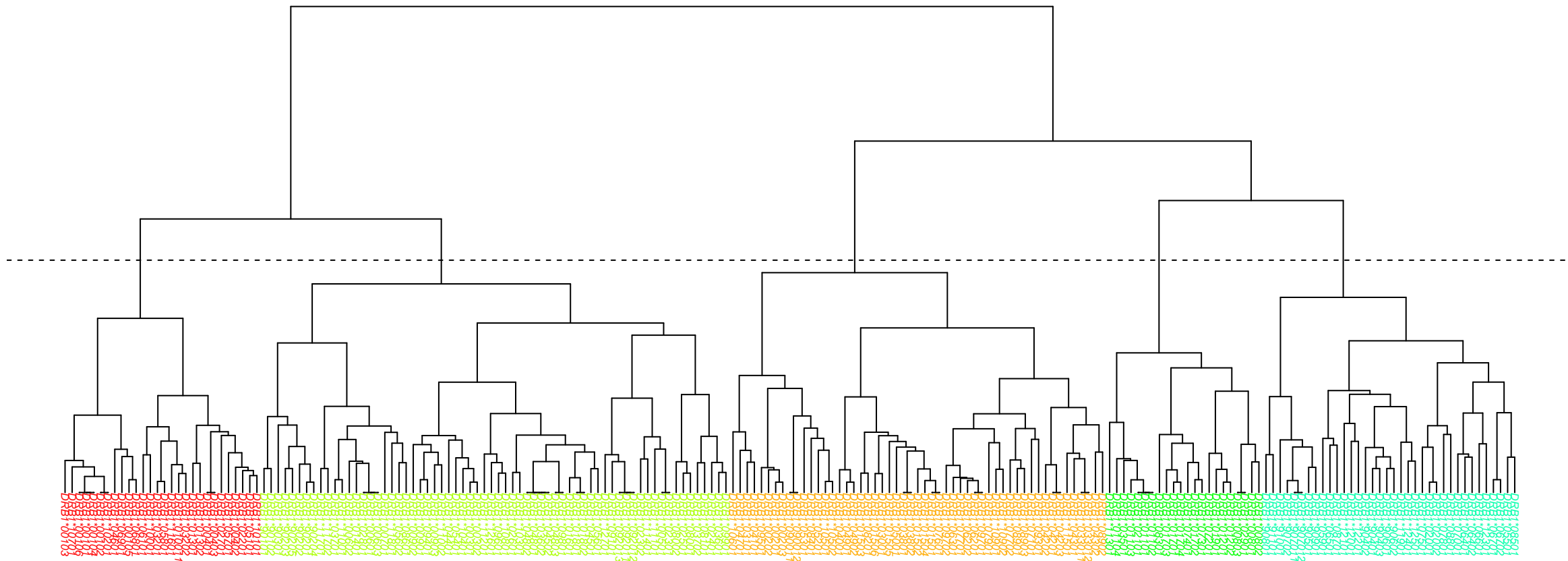

Supplement: Additional file 3: Figures S3 and S4. — Supertype clustering trees for the canine DLA-DRB1 (Figure S3) and DLA-DQB1 (Figure S4) loci with allele names included. The supertype clusters are coloured differently. (ZIP 12 kb) [file 40575_2016_38_MOESM3_ESM.zip › Supplement figS3 DRB Supertype tree.pdf]

# DLA-DQB1 supertype clustering tree

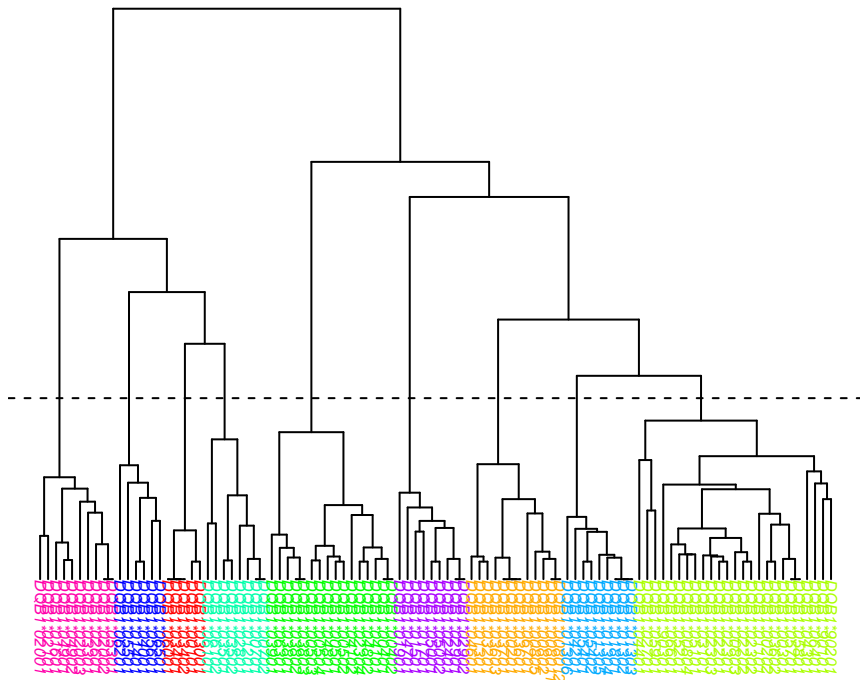

Supplement: Additional file 3: Figures S3 and S4. — Supertype clustering trees for the canine DLA-DRB1 (Figure S3) and DLA-DQB1 (Figure S4) loci with allele names included. The supertype clusters are coloured differently. (ZIP 12 kb) [file 40575_2016_38_MOESM3_ESM.zip › Supplement figS4 DQB Supertype tree.pdf]

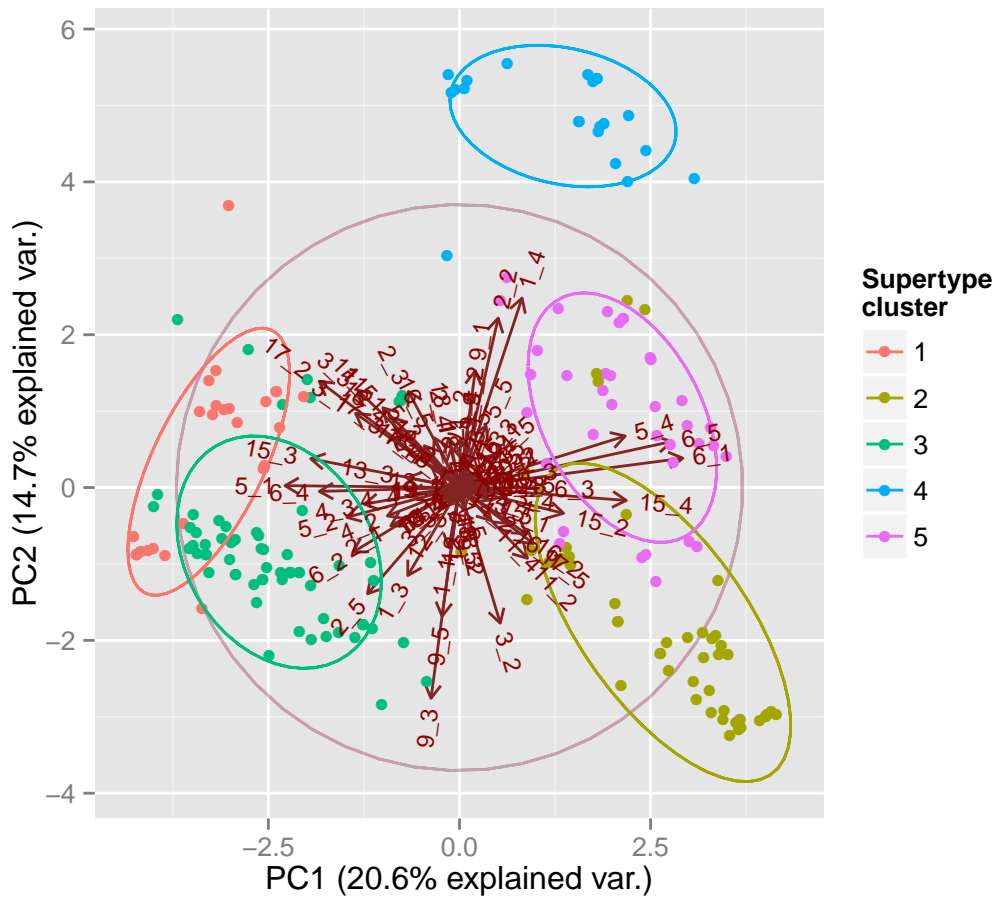

Supplement: Additional file 5: Figures S5 and S6. — Biplots of the first two PCs in supertype clusters of DLA-DRB1 (Figure S5) and DLA-DQB1 loci (Figure S6). The biplots were done using the ggbiplot function in R. Ellipses show Normal contour lines with 68% probability for each cluster. (ZIP 20 kb) [file 40575_2016_38_MOESM5_ESM.zip › Supplement figS5 PC1&PC2 DRB 5 clusters.pdf]

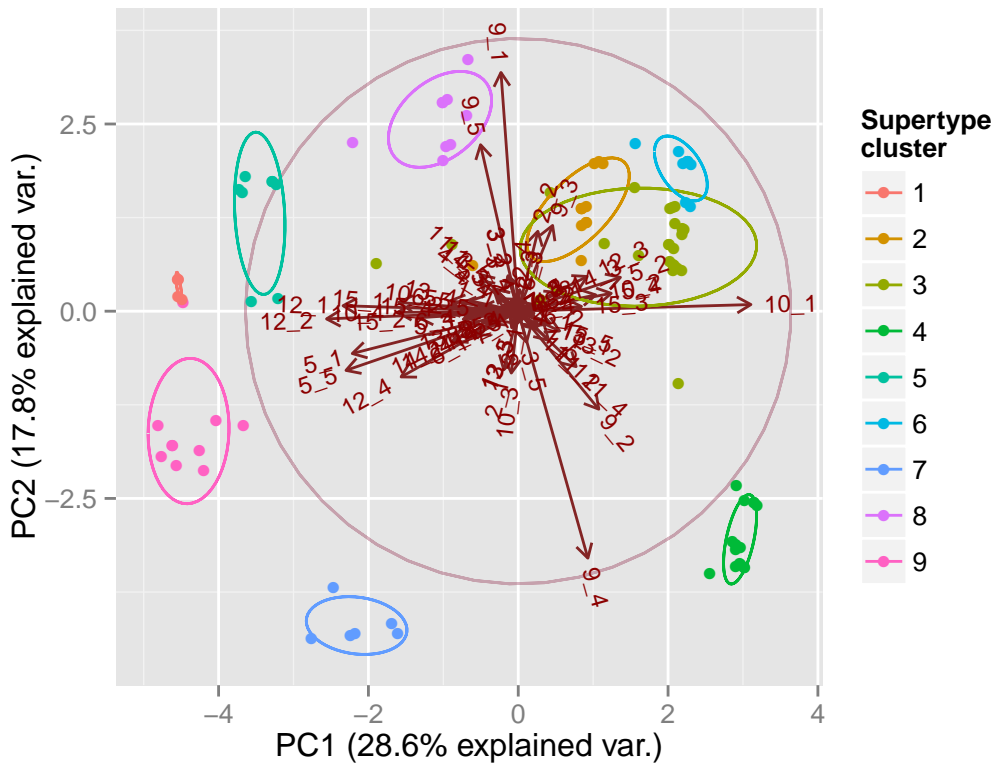

Supplement: Additional file 5: Figures S5 and S6. — Biplots of the first two PCs in supertype clusters of DLA-DRB1 (Figure S5) and DLA-DQB1 loci (Figure S6). The biplots were done using the ggbiplot function in R. Ellipses show Normal contour lines with 68% probability for each cluster. (ZIP 20 kb) [file 40575_2016_38_MOESM5_ESM.zip › Supplement figS6 PC1 & PC2 9 clusters DQB.pdf]
